# Saccade-induced temporal distortion: opposing effects of time expansion and compression

**DOI:** 10.1007/s00426-025-02116-1

**Published:** 2025-04-11

**Authors:** Lingyue Chen, Lukasz Grzeczkowski, Hermann J. Müller, Zhuanghua Shi

**Affiliations:** 1 General and Experimental Psychology, Department of Psychology, LMU Munich, Munich, Germany; 2 Graduate School of Systemic Neuroscience, LMU Munich, Munich, Germany; 3 Department of Psychology, Humboldt University of Berlin, Berlin, Germany

**Keywords:** Time distortion, Attentional gradient, Attentional blink, Chronostasis

## Abstract

Saccadic eye movements, or saccades, can distort our perception of time, as evidenced by the phenomenon of Chronostasis, where the first event after a saccade appears to last longer than it actually does. However, the impact of saccades on events following the first has never been explored. Here, we compared how participants perceived durations of first and second intervals after a saccade with their perceived durations during fixation, where no saccades occurred. We found that saccades lengthened the perceived duration of the first event, confirming Chronostasis. Moreover, when the second event occurred right after the first, its duration was perceived as shorter. Interestingly, when the second event was used as a reference, the Chronostasis effect was even stronger. Notably, this shortening of the second event persisted even when we ruled out processes like the “attentional blink” that might interfere with the timing between the two events. Our findings suggest that saccades induce a brief, uneven distribution of attentional processing in time, leading to an overestimation of the first and an underestimation of the second interval when the two intervals occur close together.

## Introduction

Subjective time is highly sensitive to various types of contextual modulation. When engrossed in reading, time seems to fly by. Actions, such as pressing a key or catching a ball, can alter the perceived timing of subsequent events. A well-known example of this phenomenon is the *stopped-clock illusion*, or *Chronostasis* (Yarrow et al., 2001). When shifting your gaze to a ticking clock, the second hand appears to freeze momentarily before moving again, making the first second after the saccade feel longer than those that follow (Knöll et al., 2013; Yarrow, 2010; Yarrow et al., 2001, 2004). In typical Chronostasis experiments, participants are presented with a digital counter displaying “0” in their visual periphery, to which they have to make a voluntary saccade. Immediately after the saccade lands on the counter, it begins incrementing (“1”, “2”,…), with each number staying on screen for a set time interval. The first interval (the “target” interval) varies, while the second interval (the ‘reference’ interval) remains constant. Participants then judge if the target interval felt longer or shorter than the reference. A widely accepted explanation for Chronostasis is that saccadic eye movements disrupt visual continuity. Since saccades cause retinal blur and induced saccadic suppression, the brain experiences uncertainty about when visual events (e.g., the onset of an interval) actually begin. To maintain perceptual stability, the brain retroactively shifts the perceived start of the event back to when the start of the saccade, creating the illusion that the first interval lasted longer than it actually did (Yarrow et al., 2004).

Actions can influence time perception not only by expanding perceived duration but also by compressing it. For instance, Morrone et al. (2005) observed that a brief 100-ms interval flashed during a saccade is perceived as shorter than its actual duration. They attributed this effect to a slowdown of the neural clock during saccade execution, a view that contrasts with the Chronostasis explanation proposed by Yarrow and colleagues (2004). Despite various efforts (e.g., Georg & Lappe, 2007; Knöll et al., 2013), no unified account has yet explained both time-expansion and compression effects. Morrone et al. noted that while “Chronostasis may be related in some way to the compression and inversion effects reported here, the connection [to saccade-induced compression] is not obvious” (Morrone et al., 2005, p. 953). To disentangle these apparently opposing effects, Knöll and colleagues (2013) systematically investigated the spatio-temporal topography of Chronostasis. They found that the overestimation of a 500-ms interval wasn’t just confined to the target location of the saccade. It also occurred for peri-saccadic events (with onset from 100 ms before to 50 ms after the saccade) at the original fixation location or positions midway on the saccadic path. Chronostasis could even be triggered by reducing stimulus visibility using - using a rapidly flipping mirror to simulate the visual effect of saccadic movement - without an actual saccade. This led Knöll et al. to argue that overestimation of time during the perisaccadic period is simply “a passive result of how the time of a stimulus onset is predicted by the visual system in general” (Knöll et al., 2013, p. 64).

Most studies on Chronostasis have focused on low-level sensory mechanisms, intentionally avoiding attentional explanations (Yarrow, 2010; Yarrow et al., 2001). In part, this avoidance stems from findings showing that Chronostasis remains largely unaffected by whether or not participants voluntarily shift their attention, in response to an arrow cue, to the target location before executing a saccade (Yarrow, 2010; Yarrow et al., 2001). However, attention and motor action are tightly and likely obligatorily coupled (Deubel & Schneider, 1996; Nobre et al., 2010; Shepherd et al., 1986). Before an action begins, attention is automatically directed toward the target location - a pattern observed in both saccadic eye movements (Deubel & Schneider, 1996; Posner, 1980; Shepherd et al., 1986) and manual pointing movements (Baldauf et al., 2006). Moreover, attention is known to distort time perception in various ways. For instance, attended events are perceived as lasting longer than unattended events of the same duration (Enns et al., 1999; Tse et al., 2004), and attention speeds up processing events near its focus, consistent with a spatial gradient of visual attention (Downing, 1988; Mangun & Hillyard, 1988). Furthermore, the first event in a sequence or an oddball event that captures attention is often perceived as longer than subsequent events (Kanai & Watanabe, 2006; Pariyadath & Eagleman, 2007; Rose & Summers, 1995).

To examine the relationship between attention and saccade-induced Chronostasis, Georg and Lappe (2007) compared time distortions at the saccade landing position versus the midway point along the saccadic trajectory. They found that Choronstasis was most pronounced at the saccade landing position, where attention was likely concentrated (cf. Deubel & Schneider, 1996), and weaker at the midway position. In contrast, Yarrow (2010) reported Chronostasis over a broader area surrounding the saccade target. By systematically varying both event onset and spatial location (saccade start, mid, and end position), Knöll et al. (2013) found that Chronostasis extended up to 50 ms after the offset of the saccade, and that this effect was similar for all locations.

While most studies have focused on time distortions of the first perisaccadic event, it remains unclear whether saccades can also distort the perception of subsequent events. Analogous to the *spatial* gradient of attention (Mangun & Hillyard, 1988), goal-directed actions may induce a *temporal* gradient where attentional resources are allocated unevenly over time. Specifically, prioritizing the first event may reduce the resources available for later events, leading to uneven time perception - a mechanism reminiscent of the attentional blink. In this classic phenomenon, participants struggle to process a second target appearing shortly after the first one (Duncan et al., 1997; Shapiro et al., 1997). This occurs because attentional and working-memory resources are still engaged with the first target, leaving fewer resources for the second. However, unlike Chronostasis, the attentional blink does not necessarily involve an overt motor action (e.g., responding to the first target may be verbal and delayed until after the end of the visual stream of target and interspersed non-target events). In Chronostasis experiments, blink-type processes may actually contribute to the overestimation of the first interval by impacting the perception of the second interval, especially when the two intervals are temporally contiguous. When the first interval ends and the second begins, attention is needed to stop timing the first interval and store it in working memory for later comparison. This attention-demanding process may interfere with the timing of the second interval, potentially causing a loss of internal clock ticks and thus leading to an underestimation of the second interval – thereby inflating the perceived duration of the first interval. A related but distinct explanation involves a saccade-induced temporal gradient of attention, where attention modulates the gate through which pacemaker pulses pass (see, e.g., the attentional gate theory of Zakay & Block, 1996a). Such a saccade-induced temporal gradient would increase attentional gating at the beginning of an event, allowing more clock ticks to be registered during the first interval (expanding its perceived duration), while reducing gating for the second interval, leading to its compression. Both the attention-blink and saccade-induced temporal-gradient accounts predict that if the reference interval for duration judgments is placed at the second temporal location, its underestimation could contribute to perisaccadic Chronostasis, causing an apparent overestimation of the duration of the first event.

Building on this framework, we conducted three experiments to investigate saccade-induced duration distortions beyond the first post-saccadic interval, with a focus on the post-saccadic second interval. To ensure comparability with standard Chronostasis studies, we largely followed the same experimental paradigm. However, Experiment 1 introduced a key manipulation: the positioning of the reference interval. The reference interval either immediately followed the test interval (No-Gap condition) or was delayed by a gap (Gap condition). We hypothesized that Chronostasis would be more pronounced in the No-Gap condition, consistent with both theoretical accounts outlined earlier. Since flash-defined intervals might potentially trigger attentional-blink effects, Experiment 2 compared flash-defined intervals with color-filled intervals, which might reduce this confound. In Experiment 3, we used color-filled intervals for better control and directly compared the first and second post-saccadic intervals to a fixed reference interval that was temporally distant from the saccade. Because this reference interval was separated in time from both the first and the second test intervals, it should not be affected by an attentional blink. However, if saccades induce an uneven temporal attentional gradient, we predicted that the second post-saccadic event would be underestimated relative to the reference interval.

## Experiment 1

### Method

#### Participants

Twenty-one healthy participants, all with normal or corrected-to-normal vision, were recruited (mean age: 26.0 years; 9 females and 12 males). The sample size was determined based on previous studies (Morrone et al., 2005; Yarrow et al., 2001), which had an average of 16 (range: 4 to 30) participants. To have sufficient power with a similar design, the sample size was increased to 21 participants. Participants were not aware of the purpose of the experiment. The study, including Experiment 1, was approved by the Ethics Committee of the LMU-Munich Faculty of Psychology and Pedagogics. Participants provided informed consent prior to the experiment, and were compensated at a rate of 9 Euro per hour for their service.

#### Apparatus

The experiment was conducted in a quiet and dark laboratory cabin. Participants sat in front of a display monitor (ViewPixx LCD, VPixx Technologies Inc.; screen refresh rate: 120 Hz), with a viewing distance of 60 cm maintained by a chinrest. Their eye movements were tracked and recorded by an EyeLink 1000 system (SR Research Ltd.), with a sampling rate of 1000 Hz. Behavioral responses were collected via a standard keyboard. The experimental program was coded in Matlab with the PsychToolbox (Brainard, 1997; Pelli, 1997) and the Eyelink toolbox (Cornelissen, Peters, & Palmer, 2002).

#### Stimuli and procedure

**Fig. 1:**
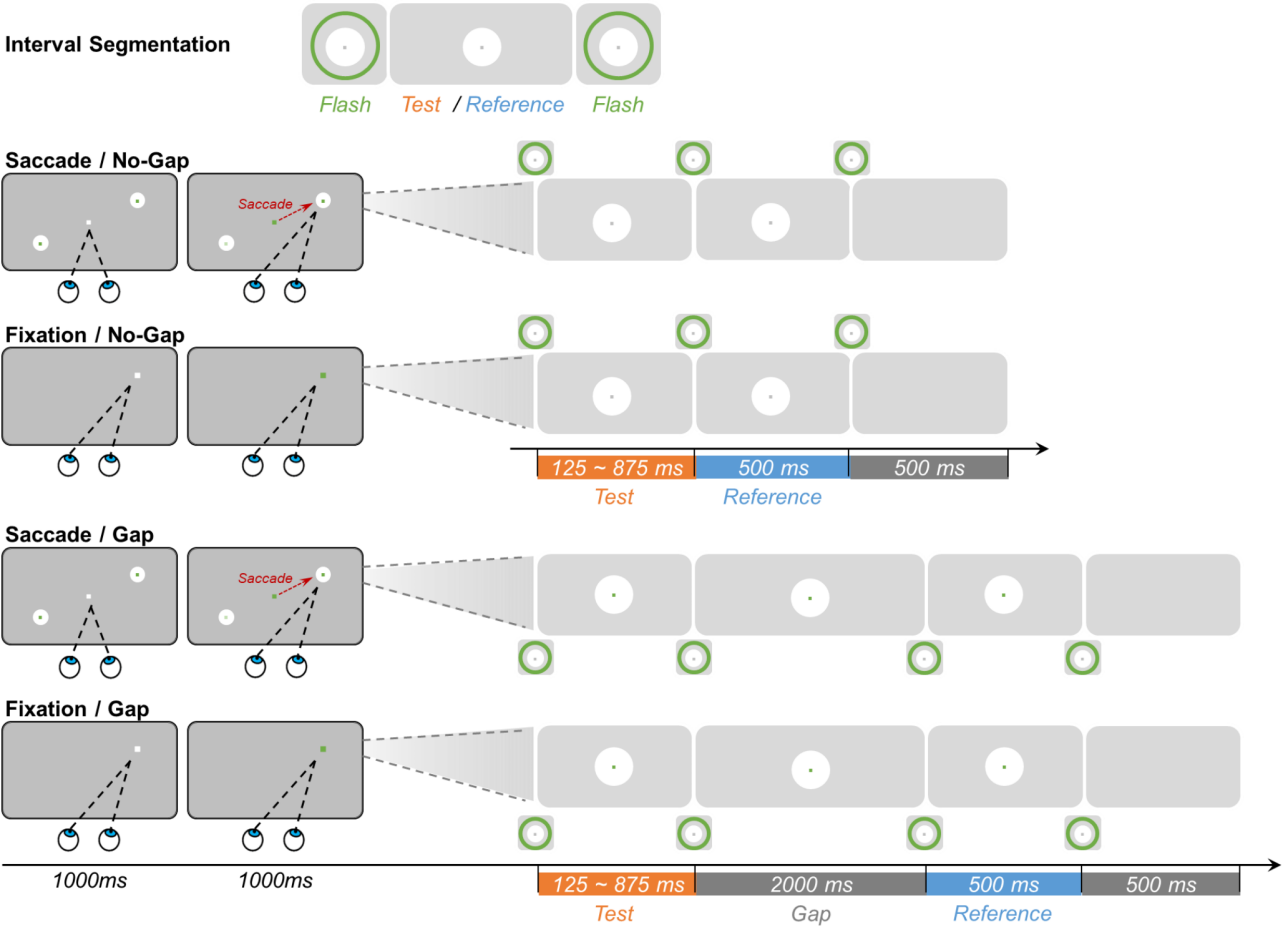
Schematic illustration of the procedure of Experiment 1. Experiment 1 comprised four blocked conditions: Saccade/No-gap, Fixation/No-gap, Saccade/Gap, and Fixation/Gap. The top row illustrates the interval segmentation for all conditions. In the bottom four rows, the left panels show the displays for the ‘*eye-movement*’ phase at the start of each trial (separately for the Saccade and Fixation conditions); and the right panels show the subsequent stimulus changes in central vision for the *interval comparison* phase (separately for the Gap and No-Gap conditions). In the Saccade conditions (1st and 3rd row), participants fixated a central white marker dot, which (after 1000 ms) turned green, cueing a saccade to one of the two peripheral disk locations. Landing on the saccade target triggered the interval-comparison phase. In the Fixation conditions (2nd and 4th row), participants fixated the dot in one corner of the screen, which turned green after one second. In the interval-comparison phase (right panels), brief (25ms) flashes of concentric green rings demarcated the test and reference intervals. In the No-Gap conditions (upper two rows), three consecutive flashes demarcated the test and reference intervals, whereas in the Gap conditions (lower two rows) four flashes demarcated the test, a gap, and the reference interval, respectively. Participants indicated which interval (the test or the reference interval) was longer by pressing a corresponding button

There were two Saccade and two Fixation conditions (see Fig. 1). In the saccade conditions, participants had to make a voluntary saccade, from a central white fixation dot (size: 0.1° of visual angle; luminance: 68 cd/m^2^), toward one of two possible locations indicated by two white disks (1°, 68 cd/m^2^). The disks were located diagonally opposite relative to the display center (randomly, either one left-down and the other right-up or, respectively, one left-up and the other right-down, at an eccentricity of 10°), and which one of the two disks the (voluntary) saccade was made to on a given trial was chosen by the participant. A trial started with the central fixation marker (on a dark gray background, 5 cd/m^2^), prompting the participant to fixate the marker for 1 s (with a spatial-error tolerance of ± 2° of visual angle). The dot then changed into green, providing the cue to execute the saccade. Once the eyes landed on the target dot, the irrelevant disk on the opposite side was extinguished immediately to minimize any potential distractions. At the chosen location, a green concentric ring around the target disk (30 cd/m^2^) was flashed for 25 ms, indicating the start of the test interval. Following a randomly varying interval (duration selected from 125, 250, 375, 500, 625, 750, and 875 ms), a second flash (of a green ring around the disk) marked the end of the test interval. In the Saccade/No-gap condition (Fig. 1, top row), the flash marking the offset of the test interval also indicated the onset of the (fixed-length) reference interval. In the Saccade/Gap condition, by contrast, a third flash was presented after a constant, 2000-ms gap to mark the onset of the reference interval (Fig. 1, third row). In all conditions, the final flash indicated the end of the reference interval. Then, after a blank interval of 500 ms, participants were prompted with the displayed question, “Which interval lasted longer: the first or the second interval?”. Participants had to make a two-alternative forced-choice (2AFC) by pressing the left or right arrow key for the first or, respectively, the second interval as having been perceived as longer. Each test interval was repeated 20 times, in random order, with the other test intervals.

To distinguish the time distortion induced by saccadic action, two baseline Fixation control conditions were introduced: one with and the other without a gap (as shown in the second and fourth row of Fig. 1). In these conditions, participants were required to fixate a single dot without making any eye movements. The position of the dot was randomly chosen from the (four) possible landing locations in the saccade conditions. Participants were instructed to maintain fixation on this location throughout the entire trial. After one second, the color of the dot changed from white to green, indicating that the test and reference intervals would ‘soon’ be presented. Similar to the Saccade conditions, a white disk appeared (with the green dot staying on in the center) after 1000 ms, roughly matching the time taken for selecting the target disk and making a saccade to it in the Saccade conditions. The subsequent sequence of events was then the same as in the saccadic conditions.

During each trial, participants’ eye movements were monitored. In the Fixation conditions, they had to keep their gaze within a specific designated area around the fixation marker (spatial error tolerance of ± 2° of radius) for the entire duration of the trial. In the Saccade conditions, they had to make the correct saccade towards the target and then maintain fixation within the designated area. Trials on which the participant blinked or fixated outside the designated area were considered invalid and immediately terminated, accompanied by a warning beep (5000 Hz, 31 Db) for 100 ms. Such failed trials (which occurred, on average, in 9.18%) were randomly retested at the end of each block to ensure that all conditions had an equal number of 140 valid trials.

Together, the experiment included four combinations of conditions based on two actions (Saccade vs. Fixation) and two reference types (No-gap vs. Gap). These conditions were tested in blocks, with the order of four blocks randomly assigned to each participant but counterbalanced across participants. Henceforth, the four conditions will be referred to as Saccade/Gap, Saccade/No-gap, Fixation/Gap, and Fixation/No-gap, respectively. The entire experiment lasted approximately two hours, with participants taking breaks between blocks as needed.

Prior to the formal experiment, participants completed two training blocks (Saccade/Gap and Fixation/No-gap conditions) to become familiar with the tasks. Each training block included 20 test intervals (100 and 1000 ms, not included in the formal test), with the standard reference interval of 500 ms. To help participants understand the task, accuracy feedback was provided at the end of each trial, in the form of a warning beep (2000 Hz, 43 Db, 100 ms) upon an incorrect response (no such accuracy feedback was provided during the format test session). The formal experiment started when the accuracy rate was above 80%, otherwise, an additional round of training was added.^1^

#### Data analysis

The ‘First’ vs. ‘Second’ responses (to the question of which of the two intervals was longer) were transformed into ‘Longer’ vs. ‘Shorter’ judgments of the test interval relative to the reference interval. The mean proportion of ‘Long’ responses for each test interval was then calculated for each condition. Psychometric curves were estimated using the R package QuickPsy (Linares & López-Moliner, 2016) for each participant in each condition, with lapse and guess rates taken into account (the mean estimated lapse rate was 0.1 in Experiment 1, which was then taken as a reference for Experiment 2). The psychometric curves allowed us to obtain two key parameters: the point of subjective equality (PSE) and the just-noticeable difference (JND). The PSE indicates the transition threshold between short and long judgments, while the JND provides an index of temporal discrimination sensitivity. Finally, these parameters were examined in repeated-measures analyses of variance (ANOVAs) with the factors Reference Timing (with vs. without Gap) and Action (Saccade vs. Fixation).

### Results and discussion

**Fig. 2:**
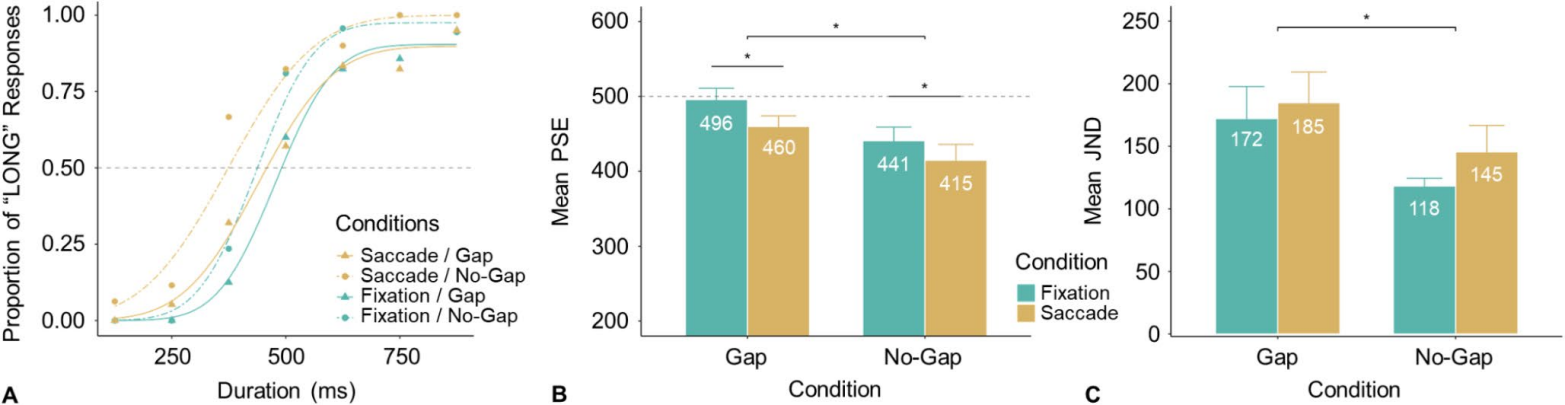
Results from Experiment 1 (**A**) Typical example of behavioral responses (dots) and fitted psychometric curves from one participant. Mean PSEs (**B**) and JNDs (**C**), and associated standard errors (SEs), for the four conditions, across all participants. The dashed horizontal line in (**B**) marks the reference interval (500 ms). The smaller the PSE, the more dilation of the test interval, as it would require a shorter (test) duration to be perceived as long as the standard (reference) duration. (*: *p* <.05)

Experiment 1 examined whether the presentation of the fixed reference interval immediately following (vs. following with a delay) the variable test interval would enhance Chronostasis. Figure 2A depicts the psychometric curves for one typical participant, with each curve representing the ratio of “long test interval” responses relative to the reference. A lower point of subjective equality (PSE) than the actual reference duration (500 ms) indicates an overestimation of the test interval, meaning that a shorter interval would be required to match the standard. The mean PSEs (with associated standard errors, ± SE) were 415 (± 21), 460 (± 14), 441 (± 18), and 496 (± 15) ms for the Saccade/No-gap, Saccade/Gap, Fixation/No-gap, and Fixation/Gap condition, respectively (Fig. 2B).

The difference between the Saccade and Fixation conditions was significant, *F*(1, 20) = 5.64, *p* =.028, $\:{\eta\:}_{g}^{2}$= 0.04, with a reduction of 31 ms for the Saccade conditions, evidencing Chronostasis (Yarrow et al., 2001). The main effect of Reference Timing was also significant, *F*(1, 20) = 6.41, *p* =.020, $\:{\eta\:}_{g}^{2}$ = 0.09: the test interval was perceived as longer when the reference immediately followed the test interval (No-gap condition) compared to when there was a 2-second gap between the two intervals (Gap condition). The Action × Reference Timing interaction was non-significant, *F* (1, 20) = 0.15, *p* =.703, $\:{\eta\:}_{g}^{2}$= 0.001.

For follow-up analysis, we conducted *t*-tests (two-tailed, adjusted for multiple comparisons) to examine whether the PSEs were smaller than the actual 500-ms reference. The results revealed the PSEs to be significantly smaller in the Saccade/Gap condition (*t*(20) = -2.83, *p* =.014, *d* = -0.63) and in both No-Gap conditions (Fixation: *t*(20) = -3.25, *p* =.008, *d* = -0.73, Saccade: *t*(20) = -4.04, *p* =.003, *d* = -0.90, however, the PSE was close to the actual 500 ms in the Fixation/Gap condition (*t*(20) = -0.28, *p* =.784, *d* = -0.06). These differences are indicative of a general tendency to (relatively) underestimate the second (i.e., reference) interval when it immediately follows the first (i.e., test) test interval.

The mean JNDs (± SE) were 145 ± 21, 185 ± 24, 118 ± 6, and 172 ± 26 ms for the Saccade/No-gap, Saccade/Gap, Fixation/No-gap, and Fixation/Gap condition, respectively (Fig. 2C). A two-way repeated-measures ANOVA revealed the main effect of Reference Timing to be significant, *F* (1, 20) = 4.59, *p* =.045, $\:{\eta\:}_{g}^{2}$ = 0.06; the main effect of Action, *F* (1, 20) = 2.47, *p* =.132, $\:{\eta\:}_{g}^{2}$ = 0.01, and the Action × Reference Timing interaction, *F* (1, 20) = 0.20, *p* =.660, $\:{\eta\:}_{g}^{2}$ = 0.00, were non-significant. Introducing a gap induced additional memory decay, worsening temporal discrimination sensitivity.

In short, Experiment 1 revealed a significant Chronostasis effect (31 ms), along with a significant effect on the temporal position of the reference interval (50 ms). When the reference interval immediately followed the test interval, the latter was perceived as longer compared to when the reference was presented after a 2-second gap. When looking at the second (reference) event, the reference interval was ‘compressed’ relative to the first interval. While this compression effect appears in part linked to the action, as evidenced by the lower PSE with vs. without a saccadic eye movement (414 ms vs. 441 ms), the main source of the compression with two contiguous intervals may be an ‘attentional blink’ – potentially exacerbated by the intervals being defined by abrupt-onset flashes: salient stimuli that engage attention automatically (Remington et al., 1992; Yantis & Jonides, 1990). That is, reinforced by the first flash, attention is allocated to and engaged by the first interval, causing a blink-type difficulty with commencing the timing of the second interval – especially given that the second flash, demarcating the transition from the first to the second interval, prompts (executive) processes directed to the first interval. Attending to the second interval may therefore be delayed (by an ‘attentional blink’), causing the second interval to be underestimated (see also the ‘attentional-gate’ account of, Zakay & Block, 1996b). In contrast, when the reference appears two seconds after the end of the first interval, attention can be reallocated to the reference event without difficulty. This avoids the attentional blink, leaving a minor saccade-induced Chronostasis effect (36 ms, based on the PSEs of 496 ms vs. 460 ms in the fixation vs. saccade conditions).

## Experiment 2

To investigate and rule out potential contributions of an attentional-blink effect, we compared flashed-defined intervals with color-filled intervals in the absence of eye-movement actions. We expected that color feature changes would generate less additional capture than abrupt flash onsets.

### Method

#### Participants

20 new healthy participants with normal color vision were recruited (mean age: 27.1 years; 12 females, 8 males). All participants were naïve to the purpose of the experiment. They provided informed consent before testing and were compensated at a rate of 9 Euro per hour. 

#### Apparatus

The experiment was conducted in the same experimental cabin using the same hardware without Eyelink as in Experiment 1.

#### Stimuli and procedure

Experiment 1 largely followed the No-Gap/Fixation condition of Experiment 1, with the following key modifications. Since saccade conditions were not included, the white disk and fixation dot were presented at the screen center. Participants were instructed to fixate on the center dot throughout the trial.

**Fig. 3:**
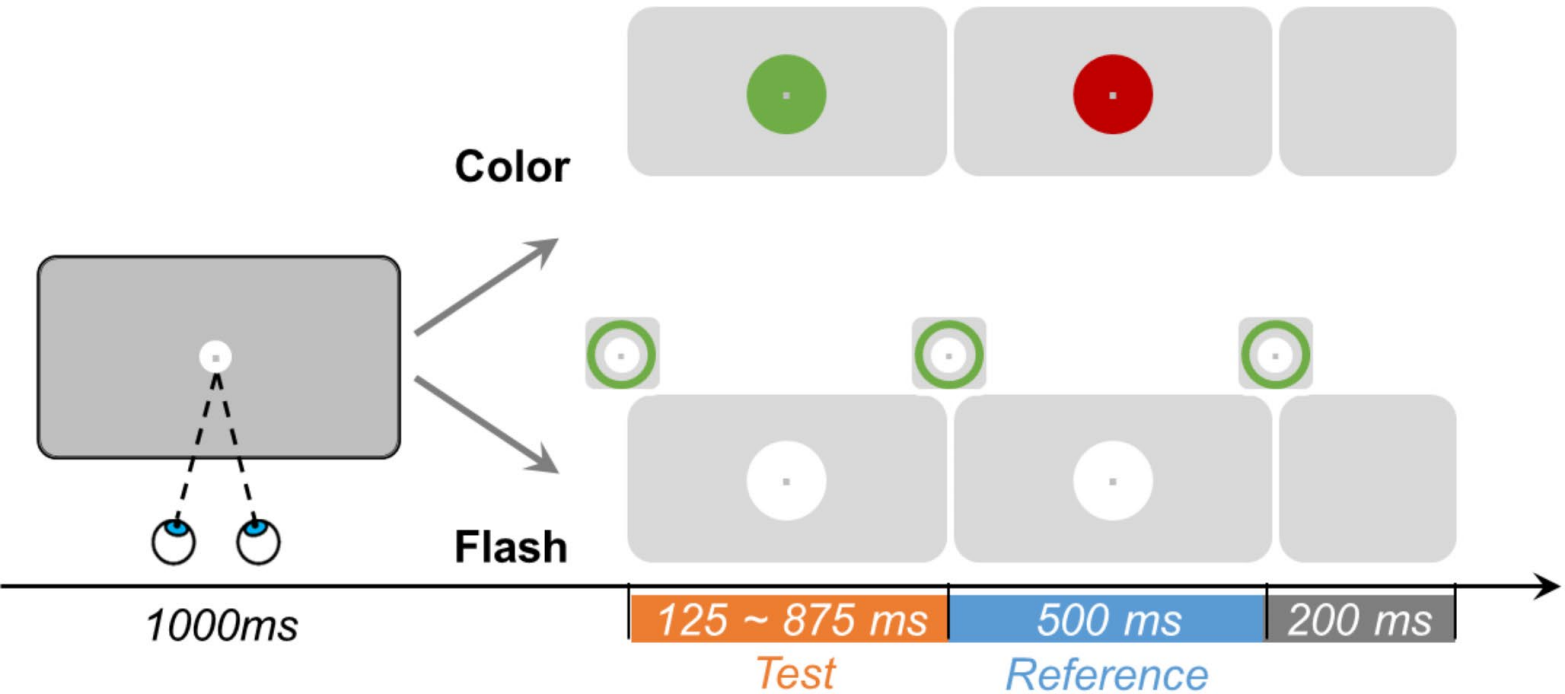
Schematic illustration of the procedure. A target disk with a center dot was displayed in the screen center throughout the trial, with participants maintaining fixation on the dot. The test and reference intervals appeared after 1000 ms fixation, which were demarcated either by a color change or a flash. In the color condition (upper row), the disk changed the color from white to another color, either to green and then to red or vice versa. In the flash condition (lower row), three concentric green flashes demarcated two intervals. The illustration on the right panel shows the stimulus changes in central vision for the interval-comparison phase. Participants compared the durations of the test (orange) and reference (blue) intervals and chose the longer one by pressing a corresponding key

The experiment featured two conditions - Flash and Color (Fig. 3, right bottom vs. upper panel) - tested in separate blocks, with block order counterbalanced across participants. In the color condition, the first (“test”) interval began when the central disk changed from white to either green (in half of the trials) or red (in the other half). The interval ended, and the second (“reference”) interval began, with a subsequent color change (red to green or green to red), with test and reference colors randomized across trials. Each of the seven test intervals was randomly repeated 20 times in both Flash and Color blocks, resulting in 140 trials per block and 280 trials total. Before formal testing in each condition, participants completed a brief training block in which test durations were either 100 or 900 ms (random order). Participants received accuracy feedback after each training trial, with a passing criterion of 80% accuracy in judging which interval (test or reference) was longer. The number of training trials was initially set to 10, but increased automatically by another 10 trials if participants failed to meet the criterion.

### Results and discussion

**Fig. 4:**
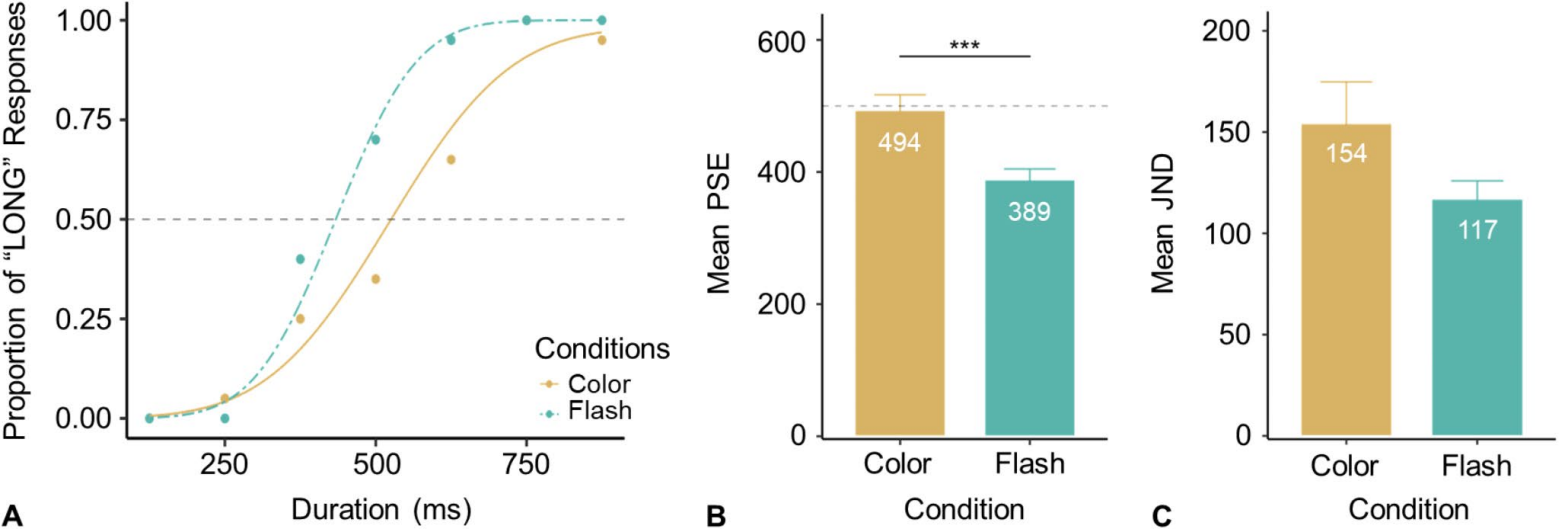
Results from Experiment 2. (**A**) A typical example of behavioral responses (dots) and fitted psychometric curves from one participant. (**B**) and (**C**) Mean PSEs and JNDs for the two conditions. The dashed horizontal line marks the reference interval (500 ms). (*: *p* <.05; **: *p* <.01, ***: *p* <.001)

To ensure high-quality data, participants’ performance was considered valid only if they correctly identified 125 ms as “short” and 875 ms as “long” (relative to the 500-ms reference interval) above 75% accuracy. Three of the 20 participants failed to meet this criterion and were excluded from further analysis. Figure 4A shows the proportion of “long” responses and the associated psychometric curves from a typical participant.

Figure 4B presents the mean PSEs (with associated standard errors): 494 (± 23) ms for the Color condition and 389 (± 16) ms for the Flash condition. A *t*-test confirmed that the PSE was significantly lower (by 105 ms) in the Flash compared to the Color condition, *t*(16) = 4.95, *p* <.001. This suggests that the attentional-blink-induced time distortion was more pronounced with flashes than with color changes. This finding also aligns with Experiment 1, where the PSE in the No-Gap/Fixation condition was shorter than the 500-ms reference duration. A simple *t*-test revealed that the attentional-blink effect was effectively eliminated when intervals were defined by color changes, as the PSE (494 ms) did not differ significantly from the 500-ms reference, *t*(16) = -0.28, *p* =.786. The mean JNDs (± SE) were 154 (± 20) for the Color condition and 117 (± 9) ms for the Flash condition. However, this difference was not statistically significant, *t*(16) = 1.74, *p* =.102, indicating comparable discrimination difficulty between the two conditions.

To summarize, Experiment 2 showed that replacing color-filled changes significantly reduced and eliminated time distortion caused by the attentional blink. Based on this finding, Experiment 3 used color-filled intervals to further investigate saccade-induced temporal distortion.

## Experiment 3

Experiment 3 was designed to directly measure action-induced distortions in the second versus the first interval following a saccadic action, with the reference interval presented 1200 ms after the second interval. If saccades induce an uneven temporal distribution of attention, similar to the attentional blink, we expected the second post-saccadic event to be underestimated relative to the reference event. To assess the robustness of this effect, we introduced slight variations from previous experiments, using a 600-ms reference interval and a 1200-ms gap.

### Method

#### Participants

21 new participants (mean age: 26.7 years; 11 females and 10 males) were recruited for Experiment 3. All had normal or corrected-to-normal vision, normal color vision, and were naïve as to the purpose of the experiment. Before participation, they provided informed consent and were compensated at a rate of 9 Euro per hour.

#### Stimuli and procedure

The setup in Experiment 3 closely followed that of Experiment 1, with the following key differences. The saccadic (target) disks were positioned 6° to the left and right of the central fixation point. Each trial started with a 500-ms central fixation marker, followed by a white arrow cue (< or >) indicating the target location. Immediately after the saccade offset, both the target and non-target disks (i.e., as a group) changed from white to either green or red, marking the onset of the first interval. At a designated time, the disk changed to the opposite color (red to green or green to red), signaling the end of the first interval and the start of the second interval. Following another designated time (see details below), the disk turned white for 1200 ms, creating a gap before the reference interval. This resulted in a gap duration of 1800 ms when the test interval was the first interval and 1200 ms when it was the second interval. Following the gap, the disk changed color (green or red) to mark the onset of the reference. The test interval was either the first or second interval, both sharing the same color as the reference interval, and session blocks were structured accordingly - either the first interval was consistently the test interval in a session, or the second interval was. Participants had to compare the test interval with the reference interval (shared the same color) and indicate which was longer by pressing the left or right arrow key.

**Fig. 5:**
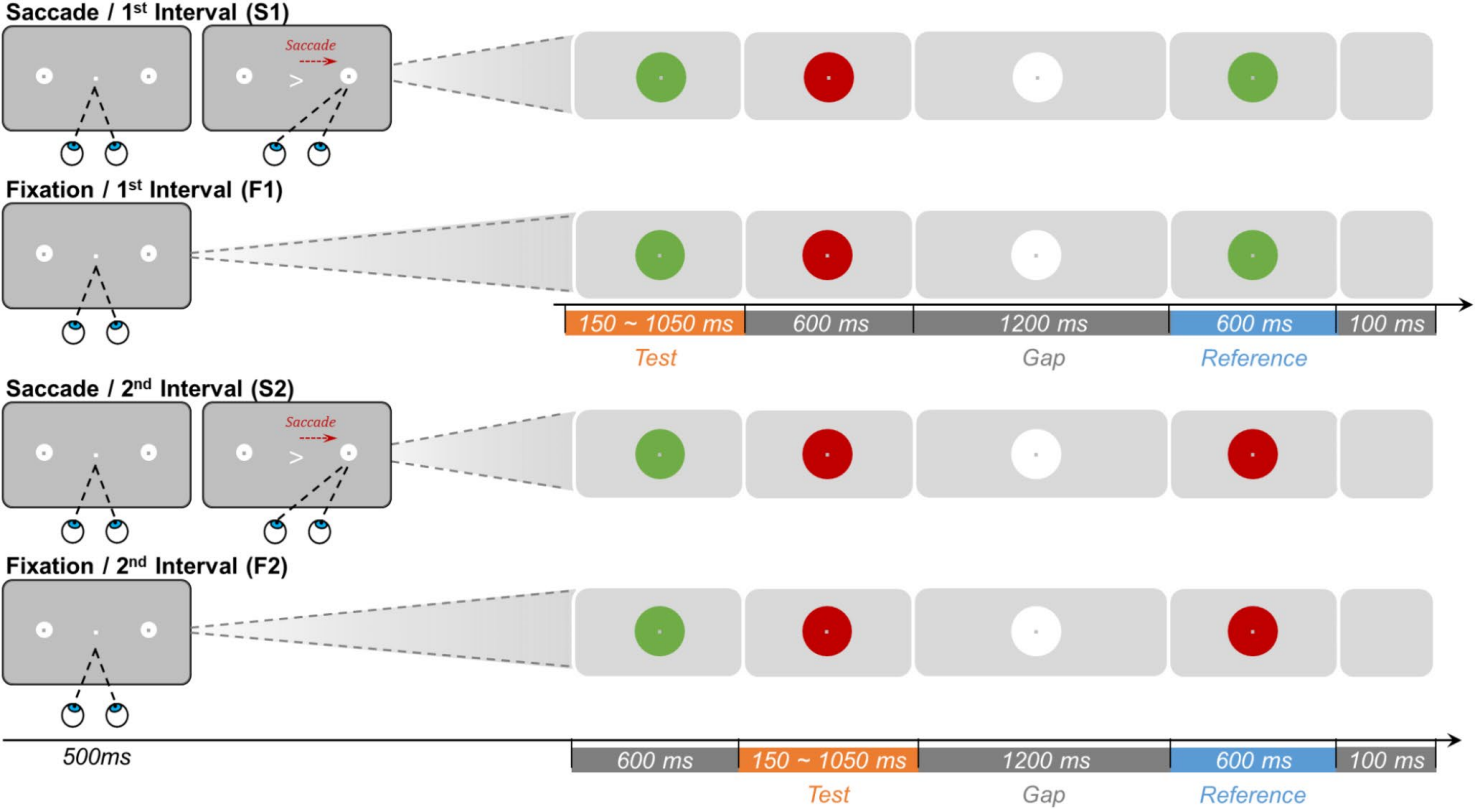
Schematic illustration of the procedure of Experiment 3. There were four blocked conditions: Saccade/1st Interval (S1), Fixation/1st Interval (F1), Saccade/2nd Interval (S2), and Fixation/2nd Interval (F2). The left panels illustrate the displays for the trial-initial action phase, separately for the Saccade and Fixation (action) conditions. The right panels depict the successive stimulus changes for the phase of interval comparison. In the Saccade conditions, participants fixated the central fixation dot for 500 ms, whereupon they were presented with a spatial arrow cue prompting them to make a saccade to the indicated target disk (1st and 3rd rows); landing on the saccade target then triggered the interval-comparison phase. In the Fixation conditions, there was no central change, requiring participants to maintain fixation in the center (2nd and 4th rows), and the interval-comparison phase began automatically after 500 ms of fixation. In the interval-comparison phase, the intervals were demarcated by changes of the disk color. In the S1 and F1 conditions (upper two rows), the first interval shared the same color with the reference interval, and so interval 1 was the task-relevant test interval (blocked per S1 and F1 session). In the S2 and F2 conditions (bottom two rows), the second interval shared the same color with the reference interval, and so interval 2 was the task-relevant test interval (blocked per S2 and F2 session). The color of the reference interval varied across conditions (green or red), while the color of the gap interval was always white. Participants indicated which interval – the test or the reference interval – was longer by pressing a corresponding key

In Experiment 3, the PSEs (and JNDs) were measured for two post-saccadic intervals: the first (S1) and the second (S2). The test and reference intervals were assigned the same color, while the non-test interval had a different color. The colors were isoluminant (25 cd/m²) and their assignment to the first and second intervals was counterbalanced across participants (i.e., half of the participants were presented with green and red as the first and second intervals in all conditions, while the other half were presented with red and green, respectively). The test interval varied randomly from 150 to 1050 ms in increments of 150 ms (7 levels), while the reference and non-test^2^ intervals were set to 600 ms (see Fig. 5).

There were also two analogous baseline conditions without eye movements, one in which the first interval was the test interval and one in which the second interval was the test interval (hereafter labeled F1 and F2, where F denotes the Fixation conditions; see Fig. 5, second and fourth rows). The procedure was identical to the saccade sessions (S1 and S2), except that observers were asked to maintain fixation on the central dot throughout the trial, ensuring that participants remained fixated at the center of the event. After 500 ms fixation, the first two intervals and the reference were presented in the same manner as in the saccade sessions.

Each participant completed all four experimental conditions (S1, S2, F1, and F2) in a random order, with the condition order counterbalanced across participants. Each session consisted of seven intervals that were randomly repeated 20 times, 10 per each side. As in Experiment 1, participants’ eye movements were monitored throughout each trial. Any trials with incorrect eye movements (on average, 12.77%) were retested in a random order at the end of each block, ensuring that all conditions had an equal number of 140 valid trials.

### Results and discussion

**Fig. 6:**
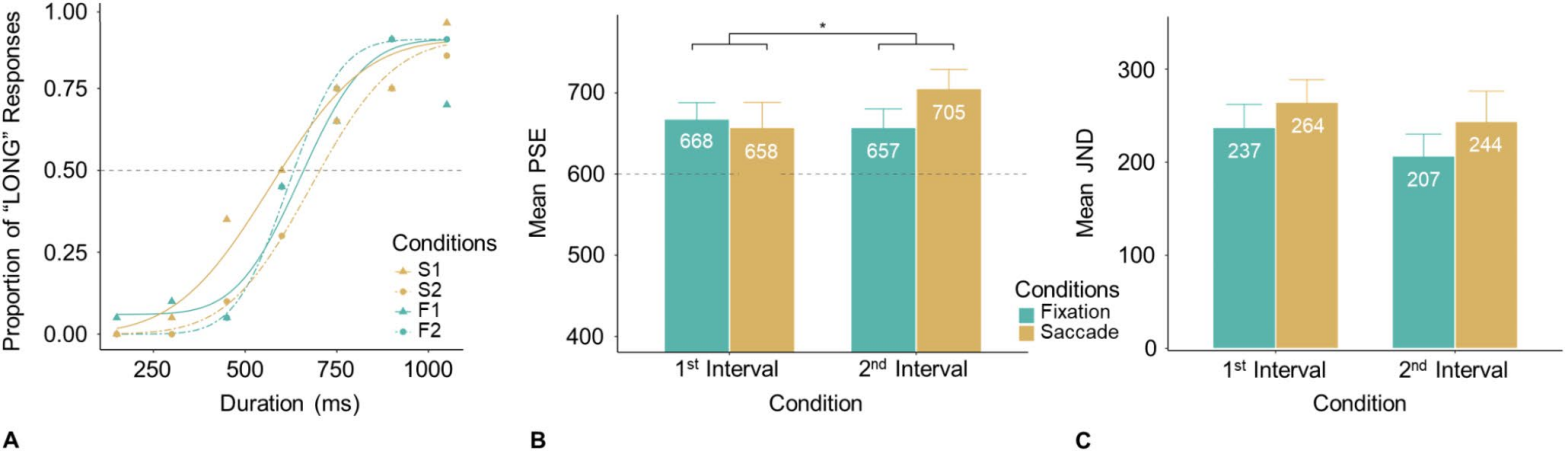
Results from Experiment 3. **(A)** A typical example of behavioral responses (dots) and fitted psychometric curves from one participant. (**B**) Mean PSEs and (**C**) JNDs for the four conditions from all participants. The dashed horizontal line in (**B**) marks the reference interval (600 ms). The increased PSE value for the second test interval in the saccade condition (compared to the other conditions) indicates compression of this interval, as it would require a longer duration for it to be perceived as long as the delayed reference interval. (*: *p* <.05)

Figure 6A depicts typical responses from one participant and associated fitted response curves, while Figure 6B and C show the average PSEs and JNDs across all participants.

The mean PSEs (± SE) for all participants were 658 ± 31 ms (S1), 705 ± 23 ms (S2), 668 ± 20 ms (F1), and 657 ± 23 ms (F2). A repeated-measures ANOVA revealed a significant Action (Saccade, Fixation) × Test-Interval (1, 2), *F*(1, 20) = 5.84, *p* =.025, $\:{\eta\:}_{g}^{2}$ = 0.02. However, the main effects were non-significant: Action, *F*(1, 20) = 1.09, *p* =.309, $\:{\eta\:}_{g}^{2}$ = 0.01; Test-Interval, *F*(1, 20) = 1.03, *p* =.323, $\:{\eta\:}_{g}^{2}$ = 0.01. The interaction was primarily driven by the ‘odd-one-out’ large PSE in the S2 condition, relative to comparable PSEs in the other three conditions. Further post-hoc comparisons revealed a significant difference between the S2 and S1 (*t*(20) = 2.00, *p* =.045, *d* = 0.45) and between the S2 and F2 (*t*(20) = 2.12, *p* =.045, *d* = 0.47), but not between the F1 and S1 (*t*(20) = -0.49, *p* =.627, *d* = -0.11). This pattern confirms that the post-saccadic second interval (S2) was greatly compressed.

In this experiment, we failed to find any significant Chronostasis. Given that the reference interval was fixed at 600 ms, we further conducted simple *t*-tests to assess absolute over- or underestimations. Results showed that all PSEs were larger than 600 ms (*p*s < 0.05), meaning that regardless of saccade presence, the *first* interval (F1 or S1) was perceived as shorter than the reference interval, even though the reference interval was separated from the end of the first interval by 1800 ms - comparable to the 2000-ms separation in Experiment 1. In other words, when the first interval was judgment-relevant, it too was compressed (relative to the delayed reference interval) to some extent. We tentatively attribute this finding to the presence of a second, irrelevant interval between the task-relevant first and the reference interval, which may have given rise to a *recency effect*, biasing perception toward the final reference interval (for further discussion, see General Discussion).

Figure 6C depicts the mean JNDs (± SE): 264 ± 24 ms (S1), 244 ± 33 ms (S2), 237 ± 25 ms (F1), and 207 ± 23 ms (F2). A two-way repeated-measures ANOVA revealed no significant effects (Action, *F*(1, 20) = 3.11, *p* =.093, $\:{\eta\:}_{g}^{2}$ = 0.02; Test-Interval, *F*(1, 20) = 1.91, *p* =.183, $\:{\eta\:}_{g}^{2}$ = 0.01; interaction, *F*(1, 20) = 0.05, *p* =.818, $\:{\eta\:}_{g}^{2}$ = 0.00).

#### Comparisons of percentage distortions

Comparisons between the Saccade and Fixation conditions revealed that saccade-induced expansion of the post-saccadic first interval (S1–F1) was minimal (1.7% distortion). In contrast, the post-saccadic second interval underwent substantial compression (S2–F2; 8.0%), similar in magnitude to the direct comparison between the two saccadic intervals (S2–S1, 8.0%). Interestingly, the percentage distortion closely matches that observed in Experiment 1, where the post-saccadic first intervals were compared with and without a gap (Saccade/Gap - Saccade/No-gap: 9.0%). This consistency highlights a key finding across both experiments: the systematic underestimation of the second interval. Experiment 1 demonstrated this when the second interval served as the reference interval, while Experiment 2 confirmed it when the second interval was the test interval. Thus, these findings indicate that saccade-induced time distortions extend beyond the first post-saccadic event, also impacting the second event.

## General discussion

Action influences how we perceive time (Merchant & Yarrow, 2016). Previous research has shown that saccadic eye movements can either expand or compress the perceived duration of the first peri-saccadic event (Morrone et al., 2005; Yarrow et al., 2001). However, much less is known about how saccades affect the perception of subsequent events. This study aimed to investigate whether and how goal-directed saccades influence the perceived duration of subsequent events, particularly that of the second post-saccadic interval, which is typically used as the reference interval in Chronostasis studies (e.g., Georg & Lappe, 2007; Park et al., 2003; Yarrow et al., 2001, 2004).

### Summary of experiments and key findings

Experiment 1 revealed that Chronostasis was stronger when the reference interval immediately followed the post-saccadic first interval (No-Gap condition), compared to when the two intervals were separated by a gap. We considered two potential explanations for this overestimation of the post-saccadic time in the No-Gap condition. First, attention may have been drawn toward the first post-saccadic interval due to the natural coupling between spatial attention and saccadic eye movements (Deubel & Schneider, 1996; Shepherd et al., 1986). Such an attentional shift could have enhanced the perceived duration of the first event, while reducing attention to the second, making the latter appear compressed by comparison. Second, the sudden onset of the first interval captures attention, a well-documented effect of abrupt visual stimuli (Remington et al., 1992; Yantis & Jonides, 1990). This may have given rise to an ‘attentional blink’ at the end of the first interval, a control-demanding point (Kawahara et al., 2006) at which the ‘clock’ had to be stopped and the result buffered in working memory. These attention-demanding processes could have delayed the start of the timing of the second interval, effectively compressing it. In contrast, in the presence of a gap between intervals, the attentional blink would have occurred during the gap, leaving the second interval’s timing unaffected.

It is important to note that an attentional-blink-like compression of the second interval, though, does not fully explain why the first interval was still overestimated when a gap was present. The persistence of the Chronostasis effect in the Gap condition suggests that additional mechanisms are involved. To clarify whether the distortions were driven by how intervals were defined, Experiment 2 examined duration perception without eye movements. The results confirmed that a blink-like effect was evident when intervals were marked by salient abrupt-onset flashes but disappeared when they were defined by isoluminant color changes.

Building on this, Experiment 3 directly tested the hypothesis that saccades induce a temporal attentional gradient, in which attention is transiently enhanced for the first post-saccadic event but reduced for the second that immediately follows. To investigate this, we treated the first and second intervals as test intervals and informed participants in advance which one they would judge in each block. The reference interval was presented after a gap following the second interval, minimizing potential interference. Additionally, we used filled color changes instead of abrupt-onset flashes to eliminate blink-induced distortions, allowing us to isolate any saccade-induced temporal-gradient effects. The results showed no significant saccade-induced Chronostasis when participants judged the first interval, with its perceived duration being similar in the Saccade and Fixation conditions. This finding does not easily square with the temporal attentional-gradient account, requiring further discussion (see below). However, when participants judged the second interval, a significant compression emerged in the saccade condition compared to the fixation control condition, consistent with the prediction by the “temporal attentional-gradient” account.

One possible reason for the absence of saccade-induced Chronostasis for the first interval may relate to the presence of a second, task-irrelevant interval intervening between the first and the reference interval. This could have triggered a recency effect, where the final reference interval dominates perception, leading to trace decay and a degree of compression for earlier intervals. Supporting this, we found that the underestimation of the first interval was similar to that of the second interval in the fixation condition. Alternatively, it is known that a group of intervals tend to be assimilated toward an ensemble mean in low-level perceptual processing (Baykan et al., 2023; Burr et al., 2013; Nakajima et al., 1992; Ren et al., 2020). If the second interval was significantly compressed, it may have biased perception of the first interval, effectively negating any minor Chronostasis effects that might otherwise have occurred, as suggested by Experiment 1. Also, as shown by Knöll et al. (2013), the Chronostasis effect can quickly disappear when the critical event is presented 50 ms after the saccade. These factors may all have contributed to the lack of saccade-induced Chronostasis in Experiment 3.

### Theoretical considerations

The present study highlights the intricate nature of subjective time distortions induced by saccades. Our results indicate that stimulus onset, saccadic action, and the timing of the reference interval all contribute to how durations are perceived. In traditional Chronostasis experiments, a digital clock is often used to display a sequence of time intervals marked by digit changes. The first digit flip after the saccade typically serves as the test interval (from 0 to 1), while the following interval acts as the reference - a setup comparable to the No-Gap condition in Experiment 1 (Georg & Lappe, 2007; Park et al., 2003; Yarrow et al., 2001).

However, as our results demonstrate, the second interval (the reference) can also be influenced by saccades, suggesting that the distortion is not confined to the first peri-saccadic event. Saccade-coupled attention may create a temporal attentional gradient, enhancing processing temporarily during the peri-saccadic period while reducing attentional resources for events immediately afterward. This was evident in Experiment 3, where the second post-saccadic interval was compressed when compared to a reference interval presented after a gap. Notably, in the fixation baseline conditions of Experiment 3, both the first and second intervals yielded comparable PSEs (668 ms and 657 ms, respectively), suggesting that without saccades, the two intervals were perceived similarly. Although both were slightly underestimated relative to the reference interval - likely due to the gap - their similarity suggests that the significant compression observed for the second post-saccadic interval was directly caused by the preceding saccadic eye movement. Unlike the first post-saccadic interval, where onset uncertainty due to the saccade might necessitate compensatory adjustments (Yarrow et al., 2001), the onset of the second interval occurred well beyond the peri-saccadic time window (600 ms after the saccade). This timing suggests that traditional explanations for Chronostasis, such as onset compensation (Yarrow et al., 2001) or low-level sensory factors (Knöll et al., 2013), cannot fully account for the compression of the second interval. It is important to acknowledge, however, that the compression of the second post-saccadic interval contributes to - but does not entirely explain - the Chronostasis effect. Previous studies have reported robust Chronostasis illusions even when the reference was temporally distant from the saccadic action, separated by a gap of 500 ms or even 1000 ms (e.g., Knöll et al., 2013; Yarrow et al., 2004).

One possible explanation for the compression of the second post-saccadic interval is an uneven spatio-temporal attentional gradient linked to saccadic eye movements. Spatially, attention is concentrated at the landing position of the saccade (i.e., the saccadic target) and gradually decreases outward from there (Mangun & Hillyard, 1988). This gradient also accounts for phenomena such as the line-motion illusion (Downing & Treisman, 1997; Hikosaka et al., 1993), in which a flash preceding the onset of a closeby line creates the illusion that the line expands outwards from the position of the flash. Temporally, planning and executing a voluntary saccade involves the preparatory shift of attention toward the target location (e.g., Deubel & Schneider, 1996; Shepherd et al., 1986), giving rise to a relatively transient post-saccadic attentional enhancement of objects or events at this location (see also Müller & Rabbitt, 1989). Thus, the first post-saccadic event occurring there would benefit from this enhancement, while the second event would fall into an attentional trough (perhaps analogous to the “inhibition-of-return” effect in the spatial domain; e.g., Klein & Ivanoff, 2008, for a review), compromising its temporal processing and leading to it being perceived as shorter than its actual duration. Overall, this account is consistent with previous findings suggesting that attention modulates the Chronostasis effect. For example, Chronostasis diminished when the peri-saccadic event was presented spatially outside the focus of attention, such as at a midway position along the saccadic trajectory (Georg & Lappe, 2007; but see Knöll et al., 2013). Our results extend this perspective, showing that saccade-induced temporal attentional modulation is not limited to the first post-saccadic event but also affects subsequent intervals. It is important to highlight that the compression effect seen in our study may differ from the saccadic compression reported in Morrone et al. (2005). The latter is restricted to short intervals, such as 100 ms, usually occurring within 200 ms after the completion of the saccade, whereas our findings regarding the second post-saccadic event (on average 500 to 600 ms) extend well beyond this timeframe.

Further work is required to substantiate this saccade-induced attentional gradient account. For instance, future studies could vary the onset timing of the first and second intervals to track the precise duration of the transient boost and the subsequent attentional trough induced by saccades. Additionally, investigating electroencephalogram phase oscillations may provide insight into the neural mechanisms underlying these temporal distortions. Oscillatory activity has been linked to attentional blink (Zauner et al., 2012) and temporal expectation (Cravo et al., 2013; Nobre & Van Ede, 2018), both of which may play a role in post-saccadic time estimation. It is also worth considering how experimental manipulations, such as using masks during fixation, might further clarify the mechanisms underlying time perception. While the current study did not include masks, prior work has demonstrated that fixation-based masking can mimic saccadic suppression, leading to perceptual effects like time compression (e.g., Zimmermann et al., 2014; Zimmermann et al., 2013; Terao et al., 2008). Future studies could explore whether masks influence time perception during fixation and saccades in similar ways, offering deeper insight into the interaction between visual suppression and temporal processing.

In conclusion, saccadic eye movements influence not only the perceived duration of the first post-saccadic event, as seen in Chronostasis, but also the subsequent events that follow. Our findings indicate that when the second post-saccadic event follows immediately upon the first event, it is subjectively compressed, amplifying the Chronostasis effect when the second event serves as a reference interval. This compression persists even when attentional-blink-like distortions are minimized or eliminated. We propose that saccades create a transient temporal attentional gradient, resulting in an overestimation of the first interval and an underestimation of the second interval immediately after the saccade.

## Data Availability

The data and materials for experiments are available at: https://github.com/msenselab/saccade-induced-temporal-grading.

## Footnotes

1 Participants who failed to pass the accuracy criterion after two rounds of training, as well as those whose eyes could not be reliably tracked by the Eyelink system (e.g., because they wore glasses), did not proceed to the formal experiment. Also, a number of participants exerted their right to quit the experiment without completing the total number of trials, stating mainly the demandingness of the task and tiredness as a reason. They were all nevertheless paid for their service at the standard rate. Overall, this led to the loss of 7 participants in Experiment 1 and 8 in Experiment 2.

2 Given this, the non-test interval could potentially also have been used as the reference interval. This is, however, unlikely, as participants were instructed (and in the practice trials trained) to compare the two *like-colored* – test and reference – intervals.
